# The Value of Biological and Conditional Factors for Staging of Patients with Resectable Pancreatic Cancer Undergoing Upfront Resection: A Nationwide Analysis

**DOI:** 10.1245/s10434-024-15070-w

**Published:** 2024-02-22

**Authors:** Thijs J. Schouten, Iris W. J. M. van Goor, Galina A. Dorland, Marc G. Besselink, Bert A. Bonsing, Koop Bosscha, Lodewijk A. A. Brosens, Olivier R. Busch, Geert A. Cirkel, Ronald M. van Dam, Sebastiaan Festen, Bas Groot Koerkamp, Erwin van der Harst, Ignace H. J. T. de Hingh, Martijn P. W. Intven, Geert Kazemier, Mike S. L. Liem, Krijn P. van Lienden, Maartje Los, Vincent E. de Meijer, Gijs A. Patijn, Jennifer M. J. Schreinemakers, Martijn W. J. Stommel, Geert Jan van Tienhoven, Robert C. Verdonk, Helena M. Verkooijen, Hjalmar C. van Santvoort, I. Quintus Molenaar, Lois A. Daamen, the Dutch Pancreatic Cancer Group

**Affiliations:** 1 Department of Surgery, Regional Academic Cancer Center Utrecht, UMC Utrecht Cancer Center and St. Antonius Hospital Nieuwegein, Utrecht University, Utrecht, The Netherlands; 2 Department of Radiation Oncology, University Medical Center Utrecht Cancer Center, Utrecht, The Netherlands; 3 Amsterdam UMC, Department of Surgery, University of Amsterdam, Amsterdam, The Netherlands; 4 Cancer Center Amsterdam, Amsterdam, The Netherlands; 5 Department of Surgery, Leiden University Medical Center, Leiden, The Netherlands; 6 Department of Surgery, Jeroen Bosch Hospital, Den Bosch, The Netherlands; 7 Department of Pathology, University Medical Center Utrecht Cancer Center, Utrecht, The Netherlands; 8 Department of Medical Oncology, Regional Academic Cancer Center Utrecht, UMC Utrecht Cancer Center and St. Antonius Hospital Nieuwegein, Utrecht, The Netherlands; 9 Department of Medical Oncology, Meander Medical Center, Amersfoort, The Netherlands; 10 Department of Surgery, Maastricht UMC+,, Maastricht, The Netherlands; 11 GROW - School for Oncology and Developmental Biology, Maastricht University, Maastricht, The Netherlands; 12 Department of General and Visceral Surgery, University Hospital Aachen, Aachen, Germany; 13 Department of Surgery, OLVG, Amsterdam, The Netherlands; 14 Department of Surgery, Erasmus MC Cancer Institute, Rotterdam, The Netherlands; 15 Department of Surgery, Maasstad Hospital, Rotterdam, The Netherlands; 16 Department of Surgery, Catharina Hospital, Eindhoven, The Netherlands; 17 Department of Surgery, Cancer Center Amsterdam, Amsterdam UMC, VU University, Amsterdam, The Netherlands; 18 Department of Surgery, Medical Spectrum Twente, Enschede, The Netherlands; 19 Department of Interventional Radiology, Regional Academic Cancer Center Utrecht, UMC Utrecht Cancer Center and St. Antonius Hospital Nieuwegein, Utrecht, The Netherlands; 20 Department of Surgery, University of Groningen and University Medical Center Groningen, Groningen, The Netherlands; 21 Department of Surgery, Isala Clinics, Zwolle, The Netherlands; 22 Department of Surgery, Amphia Hospital, Breda, The Netherlands; 23 Department of Surgery, Radboud University Medical Center, Nijmegen, The Netherlands; 24 Amsterdam UMC, Department of Radiation Oncology, location University of Amsterdam, Amsterdam, The Netherlands; 25 Department of Gastroenterology, Regional Academic Cancer Center Utrecht, UMC Utrecht Cancer Center and St. Antonius Hospital Nieuwegein, Utrecht, The Netherlands; 26 Imaging Division, University Medical Center Utrecht, Utrecht University, Utrecht, The Netherlands

**Keywords:** Pancreatic ductal adenocarcinoma, Pancreatic cancer, Biological factors, Conditional factors

## Abstract

**Background:**

Novel definitions suggest that resectability status for pancreatic ductal adenocarcinoma (PDAC) should be assessed beyond anatomical criteria, considering both biological and conditional factors. This has, however, yet to be validated on a nationwide scale. This study evaluated the prognostic value of biological and conditional factors for staging of patients with resectable PDAC.

**Patients and Methods:**

A nationwide observational cohort study was performed, including all consecutive patients who underwent upfront resection of National Comprehensive Cancer Network resectable PDAC in the Netherlands (2014–2019) with complete information on preoperative carbohydrate antigen (CA) 19-9 and Eastern Cooperative Oncology Group (ECOG) performance status. PDAC was considered biologically unfavorable (R_B+_) if CA19-9 ≥ 500 U/mL and favorable (R_B−_) otherwise. ECOG ≥ 2 was considered conditionally unfavorable (R_C+_) and favorable otherwise (R_C−_). Overall survival (OS) was assessed using Kaplan–Meier and Cox-proportional hazard analysis, presented as hazard ratios (HRs) with 95% confidence interval (CI).

**Results:**

Overall, 688 patients were analyzed with a median overall survival (OS) of 20 months (95% CI 19–23). OS was 14 months (95% CI 10 months—median not reached) in 20 R_B+C+_ patients (3%; HR 1.61, 95% CI 0.86–2.70), 13 months (95% CI 11–15) in 156 R_B+C−_ patients (23%; HR 1.86, 95% CI 1.50–2.31), and 21 months (95% CI 12–41) in 47 R_B−C+_ patients (7%; HR 1.14, 95% CI 0.80–1.62) compared with 24 months (95% CI 22–27) in 465 patients with R_B−C−_ PDAC (68%; reference).

**Conclusions:**

Survival after upfront resection of anatomically resectable PDAC is worse in patients with CA19-9 ≥ 500 U/mL, while performance status had no impact. This supports consideration of CA19-9 in preoperative staging of resectable PDAC.

**Supplementary Information:**

The online version contains supplementary material available at 10.1245/s10434-024-15070-w.

Despite the development of more effective systemic therapies, pancreatic ductal adenocarcinoma (PDAC) remains associated with a 5-year survival of about 10%.^1^ For patients with localized PDAC, pancreatic resection combined with systemic therapy is considered standard treatment.^2–4^ In contrast to formerly preferred upfront resection followed by adjuvant chemotherapy, neoadjuvant treatment has gained interest over the last decennium. In patients with borderline resectable PDAC, neoadjuvant therapy has been proven to provide survival benefits and has therefore become the recommended treatment strategy in recent years.^5–8^ For patients with primary resectable PDAC, however, definitive results of ongoing randomized controlled trials on the role of neoadjuvant treatment are awaited.^9–11^

Most definitions classify primary pancreatic tumors as resectable, borderline resectable or locally advanced on the basis of the degrees of tumor contact with major vessels, vein irregularity, and thrombosis assessed on radiological imaging.^12–14^ Internationally, the most commonly used resectability criteria are the National Comprehensive Cancer Network (NCCN) guidelines, which define pancreatic tumors as resectable in case of no arterial contact and ≤ 180° portomesenteric venous tumor contact without vein contour irregularity.^12^

Treatment recommendations of current guidelines are generally based on these anatomical criteria only.^12–14^ Nevertheless, biochemical and conditional factors are known to influence the prognosis of PDAC as well.^15–17^ Biological factors include preoperative serum carbohydrate antigen (CA)19-9 and preoperative regional lymph node metastasis, while the patients’ condition is reflected by the Eastern Cooperative Oncology Group (ECOG) performance status.^18^ Recently, the International Association of Pancreatology (IAP) has proposed to expand the preoperative staging criteria by redefining borderline resectable PDAC with biological and conditional criteria, suggesting that resectability status should be assessed beyond the anatomic relationship between tumor and vessels.^19^ This has, however, yet to be validated for patients undergoing upfront resection in a nationwide setting.

Therefore, the aim of this study was to evaluate the prognostic value of biological and conditional factors for staging patients with primary resectable pancreatic cancer.

## Patients and Methods

### Study Design

A nationwide observational cohort study was performed in all 16 Dutch centers for pancreatic cancer surgery. The Strengthening the Reporting of Observational Studies in Epidemiology (STROBE) guidelines for reporting observational studies were followed.^20^ Patients who underwent upfront resection of histologically proven, primary resectable PDAC according to the NCCN criteria between 2014 and 2019 were identified from the mandatory Dutch Pancreatic Cancer Audit (DPCA).^21^ Patients with an unknown resectability status were excluded. During the study period, the recommended treatment strategy for patients with resectable PDAC was upfront resection. Neoadjuvant treatment was only administered in the context of randomized trials.^5,9^ Therefore, patients who received neoadjuvant treatment were excluded from this study.

### Data Collection

Prospective baseline characteristics and perioperative data were retrieved from the audit database after approval of the DPCG scientific committee. Additionally, detailed data on adjuvant therapy, follow-up, and survival were retrieved from patients’ medical records. Data on ethnicity and race of patients were not obtained, as these data are not available in the DPCA.^21^

For each patient, anatomical resectability status was determined retrospectively according to NCCN criteria.^12^ Subsequently, patients with primary resectable (R) PDAC were categorized on the basis of biological and conditional factors. Patients were considered to have biologically unfavorable PDAC (R_B+_) when preoperative serum CA19-9 level was ≥ 500 U/mL. Patients with serum CA19-9 levels < 500 U/mL were considered R_B−_. Patients were deemed to have conditionally unfavorable PDAC (R_C+_) if their baseline ECOG performance status was ≥ 2, and patients with an ECOG performance status of 0–1 were classified as having R_C−_ PDAC.^17,19^ Based on these criteria, patients were stratified into one of four groups, having either (1) R_B+C+_ PDAC; (2) R_B+C−_ PDAC; (3) R_B−C+_ PDAC or (4) R_B−C−_ PDAC. The serum CA19-9 level closest to the date of resection was used if multiple preoperative serum CA19-9 samples were available.

### Outcomes

The primary outcome was overall survival (OS), defined as the time between the date of tumor resection until the date of death from any cause. The secondary outcome was disease-free survival (DFS), defined as the time between the date of tumor resection until the date of PDAC recurrence diagnosis. PDAC recurrence was pathologically proven, or suspected through imaging, and preferably confirmed by consensus during a multidisciplinary meeting. Alive patients were censored at the date of last follow-up.

### Statistical Analysis

Statistical analysis was performed including only patients with complete information on “key variables,” i.e., preoperative serum CA19-9 and ECOG performance status. To assess potential selection bias resulting from the complete case analysis, baseline characteristics of included patients were compared with baseline characteristics of patients who were excluded owing to missing data. Other missing baseline data were considered missing at random and imputed based on a Markov Chain Monte Carlo method (five imputations; ten iterations).^22^ The original cohort and cohort after multiple imputations were compared for inconsistencies. Descriptive statistics were used to compare the prespecified groups. Categorical variables were presented as frequencies and compared using the chi-squared or Fisher’s exact test. Parametric continuous variables were shown as mean with standard deviation (SD) and compared using one-way analysis of variance (ANOVA). Non-parametric continuous variables were reported as median with interquartile range (IQR) and compared using the Kruskal–Wallis test. Kaplan–Meier survival curves were used to assess OS and DFS for each group, and univariate Cox proportional hazard analyses were performed to calculate survival differences. Results were presented as hazard ratio (HR) with 95% confidence interval (CI).

Several sensitivity analyses were performed to assess robustness of findings when accounting for underlying factors that may affect the results. First, the primary analysis was stratified for presence of hyperbilirubinemia (defined as preoperative total bilirubin serum levels < 20 μmol/L), considering that serum CA19-9 levels may be inaccurate in cases of hyperbilirubinemia.^23–25^ Second, a sensitivity analysis was conducted in a subset of patients with serum CA19-9 levels ≥ 5 U/mL, excluding patients who are considered non or low-secretors of CA19-9.^26,27^ A third sensitivity analysis was done using a lower CA19-9 threshold for R_B+_ PDAC, i.e., 200 U/mL, based on the results of a recent study.^16^ Furthermore, a fourth sensitivity analysis was performed using a lower ECOG threshold, i.e., ≥ 1, to define R_C+_ PDAC.

Finally, multivariable Cox proportional hazard analyses were conducted to investigate the association between serum CA19-9 and OS, and ECOG performance status and OS, adjusted for potential confounders (i.e., age, sex, tumor size, nodal stage, resection margin status, tumor differentiation, and perineural invasion).

Statistical analyses were performed using R version 3.5.1 (Bell Laboratories, NH, USA) using the “mice” and “survival” packages. A two-tailed *P* value of ≤ 0.05 was considered statistically significant.

## Results

### Study Population

**Fig. 1:**
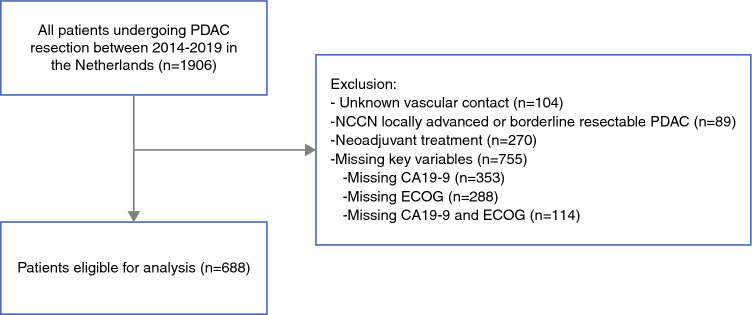
Flowchart of patient selection. *CA19-9* carbohydrate antigen 19-9, *ECOG* Eastern Cooperative Oncology Group, *NCCN* National Comprehensive Cancer Network, *PDAC* pancreatic ductal adenocarcinoma

Overall, 1906 patients were identified, of whom 1443 underwent upfront resection of NCCN resectable PDAC. Of those, 688 patients were included (Fig. 1). No differences in baseline characteristics were observed in patients who were excluded owing to missing “key variables” compared with included patients (Supplementary Digital Content 1).

Median follow-up was 31 months (IQR 20–46 months), median OS was 20 months (95% CI 19–23 months) and median DFS was 15 months (95% CI 14–17 months). The first sites of recurrence were local (22%), liver (15%), and multiple (54%). Stratification of the cohort resulted in 20 patients (3%) with R_B+C+_ PDAC, 156 patients (23%) with R_B+C−_ PDAC, 47 patients (7%) with R_B−C+_ PDAC, and 465 patients (68%) with R_B−C−_ PDAC (Table 1). Groups differed significantly with regard to age ASA III–IV, BMI, CCI score, serum bilirubin levels, vascular resection, hospital stay, pathologically measured tumor size, nodal stage, resection margin status, and adjuvant chemotherapy.

**Table 1:** Baseline characteristics of 688 patients undergoing resection of anatomical resectable PDAC according to the predefined subgroups

Characteristic	R_B+C+_^a^ (*n* = 20)	R_B+C−_ (*n* = 156)	R_B-C+_ (*n* = 47)	R_B-C−_ (*n* = 465)	*P* value
Male sex, no. (%)	14 (70)	85 (55)	22 (47)	243 (52)	0.35
Age in years, median (IQR)	69 (64–74)	70 (63–75)	73 (66–77)	68 (60–74)	**< 0.001**
BMI, median (IQR)	26 (22–28)	24 (22–27)	25 (21–28)	24 (22–27)	**0.02**
Charlson Comorbidity Index, no. (%)					**< 0.01**
< 2	9 (45)	113 (73)	22 (47)	306 (66)	
≥ 2	11 (55)	43 (27)	25 (53)	159 (34)	
ASA classification, no. (%)					**< 0.001**
I–II	7 (35)	112 (72)	24 (51)	358 (77)	
III–IV	13 (65)	44 (28)	23 (49)	107 (23)	
ECOG performance status, no. (%)					
0–1	0 (0)	156 (100)	0 (0)	465 (100)	**< 0.001**
2–4	20	0 (0)	47 (100)	0 (0)	
Serum bilirubin (Umol/L), median (IQR)	26 (8–66)	64 (17–172)	19 (10–74)	22 (9–108)	**< 0.001**
Serum CA19-9 (U/mL), median (IQR)	1500 (1018–3392)	1195 (788–2334)	63 (20–168)	91 (26–228)	**< 0.001**
Type of surgery, no. (%)					**0.03**
Open	16 (80)	137 (88)	45 (96)	365 (78)	
Laparoscopic	2 (10)	8 (5)	2 (4)	55 (12)	
Robotic	2 (10)	11 (7)	0 (0)	45 (10)	
Type of resection, no. (%)					0.41
Pancreatoduodenectomy	15 (75)	137 (88)	36 (76)	378 (81)	
Distal pancreatectomy	4 (20)	16 (10)	9 (19)	76 (16)	
Total pancreatectomy	1 (5)	3 (2)	2 (5)	11 (2)	
Vascular resection, no. (%)	5 (25)	50 (32)	13 (28)	97 (21)	**0.04**
Major postoperative complications, no. (%)^b^	6 (30)	73 (47)	21 (45)	175 (38)	0.16
Hospital stay in days, median (IQR)	11 (8–19)	11 (8–17)	13 (9–17)	10 (7–16)	**< 0.001**
30-day mortality after surgery owing to complications (%)	0 (0)	2 (1)	2 (4)	8 (2)	0.52
Pathologically measured tumor size in cm, no. (%)					**< 0.01**
≤ 2 cm	3 (10)	10 (6)	9 (19)	92 (20)	
> 2 cm to ≤ 4 cm	13 (65)	109 (70)	30 (63)	296 (64)	
> 4 cm	4 (25)	37 (24)	8 (17)	77 (17)	
8^th^ AJCC N stage, no. (%)					**0.03**
N0	2 (10)	32 (21)	12 (26)	132 (28)	
N1	7 (35)	60 (38)	20 (41)	198 (42)	
N2	11 (55)	64 (41)	15 (33)	136 (29)	
Lymphovascular invasion, no. (%)	14 (70)	99 (64)	28 (60)	289 (63)	0.86
Perineural invasion, no. (%)	19 (95)	140 (90)	42 (89)	391 (84)	0.21
Resection margin status, no. (%)					**< 0.01**
R0 ≥ 1 mm	13 (65)	63 (40)	20 (42)	255 (55)	
R1 < 1 mm	7 (35)	93 (60)	27 (58)	210 (45)	
Tumor differentiation, no. (%)					0.81
Well/moderate	15 (75)	113 (73)	33 (70)	351 (75)	
Poor	5 (25)	43 (27)	14 (30)	114 (25)	
Adjuvant chemotherapy	8 (40)	88 (57)	22 (47)	310 (67)	**< 0.01**

Percentages may not sum to 100% because of rounding

^a^_B+_ was defined as preoperative serum CA19-9 levels ≥ 500 U/mL, and _B−_ as preoperative serum CA19-9 levels < 500 U/mL; _C+_ was defined as an Eastern Cooperative Oncology Group performance status ≥ 2, and _C−_ as Eastern Cooperative Oncology Group performance status 0–1

^b^Major complications were defined as complications requiring a surgical or radiological intervention, intensive care unit admittance, organ failure, or death

*AJCC* American Joint Committee on Cancer, *ASA* American Society of Anesthesiologists, *BMI* body mass index, *ECOG* Eastern Cooperative Oncology Group, *IQR* interquartile range, *PDAC* pancreatic ductal adenocarcinoma

### Survival

**Fig. 2:**
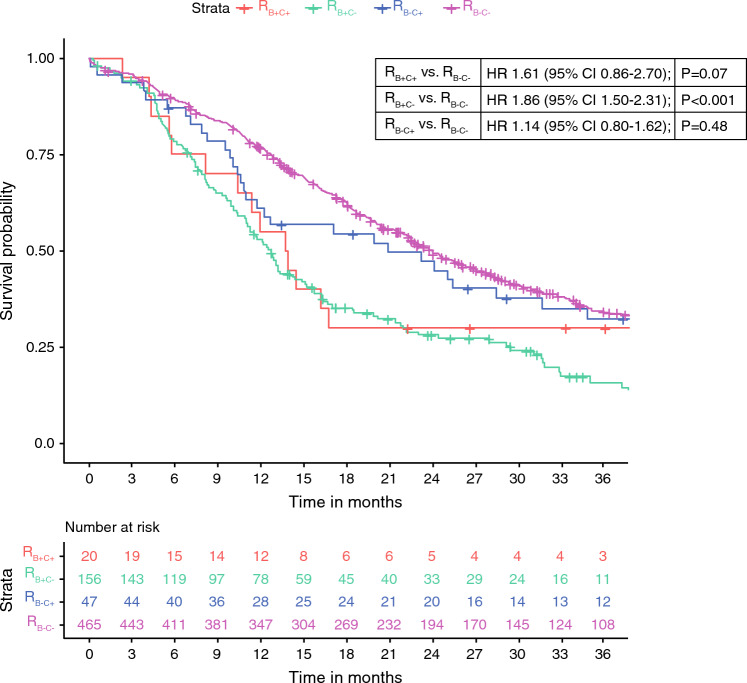
Kaplan–Meier curves and results from Cox proportional hazard analysis comparing overall survival between patients with R_B+C+_, R_B+C−_, R_B−C+_, and R_B−C−_ PDAC; R_B+_ was defined as preoperative serum CA19-9 levels ≥ 500 U/mL and R_B−_ as CA19-9 < 500 U/mL; R_C+_ was considered with an ECOG performance status ≥ 2 and R_C−_ with ECOG 0–1

Patients classified as having R_B+C+_ PDAC had a median OS of 14 months (95% CI 10 months—median not reached). Median OS was 13 months (95% CI 11–15 months) for patients with R_B+C–_ PDAC, 21 months (95% CI 12–41 months) for patients with R_B–C+_ PDAC, and 24 months (95% CI 22–27 months) for patients with R_B–C−_ PDAC (Fig. 2). Compared with R_B–C–_ PDAC (reference), this resulted in a HR of 1.61 (95% CI 0.86–2.70; *P* = 0.07) for patients with R_B+C+_ PDAC, a HR of 1.86 (95% CI 1.50–2.31; *P* < 0.001) for patients with R_B+C−_ PDAC, and a HR of 1.14 (95% CI 0.80–1.62; *P* = 0.48) for patients with R_B–C+_ PDAC.

### Sensitivity Analysis

Stratification for presence of hyperbilirubinemia identified 386 patients (56%) with hyperbilirubinemia and 302 patients (44%) without hyperbilirubinemia. In both patients with and without hyperbilirubinemia, the R_B+C–_ group had the lowest survival of all groups, being 13 months (95% CI 11–16 months), and 11 months (95% CI 10–22 months), respectively (Supplementary Digital Content 2). Results remained the same when excluding non-secretors of CA19-9 (*n* = 44; 6%) (Supplementary Digital Content 3).

**Fig. 3:**
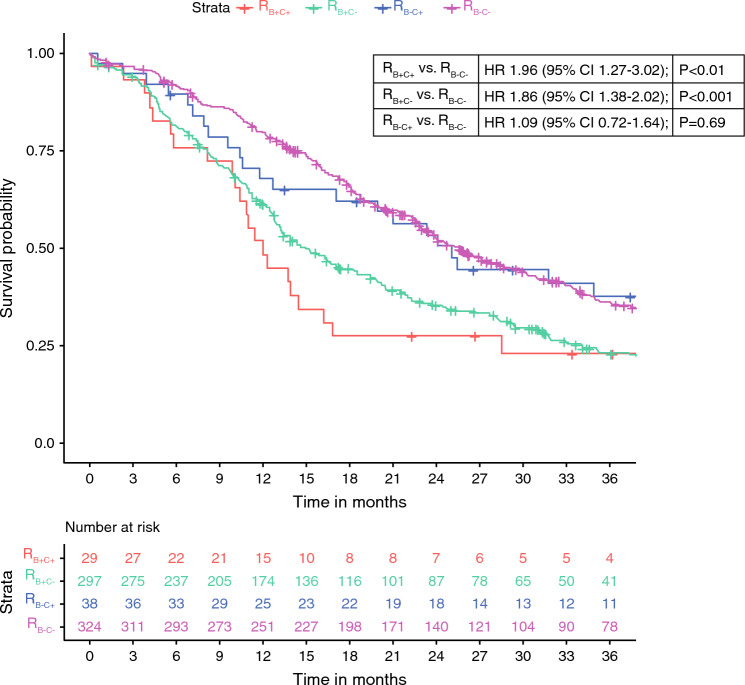
Sensitivity analysis: Kaplan–Meier curves and Cox-proportional hazard analysis comparing overall survival between patients with R_B+C+_, R_B+C−_, R_B−C+_, and R_B−C−_ PDAC, defining R_B+_ as CA19-9 ≥ 200 U/mL; R_B+_ was defined as preoperative serum CA19-9 levels ≥ 200 U/mL and R_B−_ as CA19-9 < 200 U/mL; R_C+_ was considered with an ECOG performance status ≥ 2 and R_C−_ with ECOG 0–1

A lower serum CA19-9 threshold of ≥ 200 U/mL for defining R_B+_ resulted in more patients being staged as having R_B+C+_ PDAC (*n* = 29, 4%), and R_B+C–_ PDAC (*n* = 297, 43%). Similar survival differences were found between reclassified groups, with R_B+C+_ PDAC having the worst median OS of 12 months (95% CI 10–17 months; Fig. 3). A lower threshold for R_C+_ (ECOG performance status ≥ 1; Supplementary Digital Content 4) resulted in a larger number of patients considered R_B+C+_ (*n* = 100; 15%), while the number of patients in the R_B–C+_ (*n* = 248; 36%) group also increased. Interestingly, survival was now lowest for patients in the R_B+C+_ PDAC group, with a median OS of 12 months (95% CI 10–14 months).

### Association Between Biological Factors, Conditional Factors, and OS

Multivariable analysis identified a preoperative CA19-9 serum level ≥ 500 U/mL [HR 1.62 (95% CI 1.31–1.99);* P* < 0.001] to be associated with OS. A lower CA19-9 threshold of ≥ 200 U/mL showed a similar association [HR 1.53 (95% CI 1.27–1.84);* P* < 0.001]. Worse ECOG performance status (≥ 2 versus 0–1) was not associated with OS [HR 0.99 (95% CI 0.72–1.33); *P* = 0.94]. This remained the same when lowering the ECOG threshold [≥ 1 versus 0; HR 1.08 (95% CI 0.90–1.31); *P* = 0.39].

## Discussion

This study showed that survival after upfront resection is significantly worse for patients with anatomically resectable but biologically unfavorable PDAC (serum CA19-9 ≥ 500 U/mL) than for patients with resectable PDAC and serum CA19-9 < 500 U/mL. ECOG performance status did not impact survival. Similar survival differences were found when applying a lower preoperative serum CA19-9 threshold (≥ 200 U/mL), affecting an even larger group of patients. These findings suggest that serum CA19-9 levels are valuable for preoperative staging of patients with resectable PDAC.

The resectability definition for localized PDAC is traditionally based on vascular tumor involvement. This definition was introduced to identify patients with LAPC or borderline resectable disease who have a higher risk of R1 resections, in which initial systemic treatment instead of upfront resection is recommended.^28^ Currently, most guidelines regarding treatment strategies of PDAC focus on anatomical criteria only, although the value of biological and conditional factors is increasingly emphasized.^18,29^ Considering that accurate preoperative staging of PDAC patients is relevant for guiding treatment strategies and supporting shared-decision making, evaluation of the additional value of these factors is of great importance.^30^

Previously, two smaller single-center studies were published to validate the IAP proposal, including both biological and conditional factors.^31,32^ Hayasaki et al. studied 285 patients who received preoperative neoadjuvant chemoradiotherapy, reflecting a different patient group than patients who underwent upfront resection in our study.^32^ The study by Kato et al. included only 12 conditionally unfavorable patients, impeding a proper analysis of the value of conditional factors.^31^ The current study is the first to validate the IAP proposal in a large, nationwide cohort of patients who underwent upfront resection. This resulted in a larger number of patients available for analysis in each subgroup, enhancing the power of findings. Moreover, in contrast to previous studies, this study also investigates the interplay of biological and conditional factors, providing a deeper understanding.

An important finding of this study was that, despite having anatomically resectable PDAC, patients with R_B+_ disease have a dismal prognosis when compared with patients with R_B–_ disease. After correction for potential inaccuracy of CA19-9 owing to hyperbilirubinemia and after exclusion of non or low-secretors of CA19-9, survival differences remained at a similar disadvantage for the R_B+_ PDAC group. This supports the importance of tumor biology to stage patients beyond anatomical resectability criteria, as also underlined by recent studies demonstrating lower survival rates in patients with biologically unfavorable PDAC.^31–35^ Nevertheless, although previous studies showed a strong association between preoperative serum CA 19-9 levels and survival, most international guidelines do not incorporate CA19-9 in the treatment recommendations for localized PDAC.^16,23,31–37^ One study determined CA19-9 ≥ 200 U/mL to be associated with worse survival outcomes after PDAC resection, while another study established the threshold of CA19-9 at 1000 U/mL.^16,31^ Considering that a lower cutoff for CA19-9 may impact a higher number of patients, a sensitivity analysis was performed with a less strict threshold of CA19-9 ≥ 200 U/mL for R_B+_. Survival outcomes after upfront resection for the larger group of R_B+_ patients with a serum CA 19-9 ≥ 200 U/mL were significantly worse compared with the other PDAC groups. Therefore, a preoperative serum CA19-9 threshold of ≥ 200 U/mL may be considered for preoperative staging of resectable PDAC.

In contrast to the widely studied importance of preoperative serum CA19-9, less is known about the need to incorporate conditional factors for preoperative staging of resectable PDAC. Conditional factors might be important, as they are negatively associated with complications after surgery, refraining from chemotherapy, and poor survival.^17,38^ Previous studies reported ECOG performance status to be a major prognostic factor for survival in patients with PDAC.^17,31,32,38^ Moreover, other factors reflecting conditional status have also been associated with survival, such as radiomics and body composition measures on preoperative imaging.^39,40^ Nevertheless, only the ECOG performance status was included in proposed staging criteria. In our study, however, survival in patients with R_B–C+_ PDAC was similar to survival in R_B–C–_ patients. For the small group of patients with R_B+C+_, survival outcomes seemed mainly disadvantaged because of an unfavorable tumor biology. Furthermore, ECOG performance status was not associated with decreased OS in a multivariable model. Nevertheless, patients with a poor performance status might have been determined eligible for surgery only after careful selection during multidisciplinary team meetings. These meetings have been initiated to screen patients on frailty and surgical risk, herewith improving patient selection while also paying attention to prehabilitation to improve patient fitness before surgery.^41^ Consequently, failure to demonstrate a difference in survival between R_B–C+_ patients and R_B–C–_ might be a result of confounding by indication.

The addition of biological and conditional parameters for preoperative staging has been proposed previously and usually contained elevated preoperative serum CA19-9.^18,29,42^ The IAP consensus statement regarding novel borderline resectability criteria also considered patients with preoperative regional lymph nodes metastasis, diagnosed by positron emission tomography-computed tomography (PET-CT) or nodal biopsy, to have borderline resectable disease.^19^ The value of preoperative regional lymph node metastasis as an expression of biologically unfavorable PDAC could not be assessed in this study since PET-CT and nodal biopsy were not performed routinely. Interestingly, however, higher rates of positive pathological lymph nodes were found for patients with R_B+_. Evaluation of the additive value of biopsy or PET-CT to assess preoperative regional lymph node metastasis could therefore be a potential focus of future studies.

Currently, guidelines recommend administration of neoadjuvant chemo(radio)therapy for anatomically borderline resectable PDAC, supported by the results of recent randomized controlled trials.^43^ These studies showed better OS after neoadjuvant treatment in borderline resectable patients compared with upfront surgery. Since R_B+_ patients had a dismal prognosis after upfront tumor resection in our study, neoadjuvant treatment with intensive chemotherapeutic regimens, such as FOLFORINOX, could be suggested for this group. This has already been implemented in clinical practice in some large-volume pancreatic expert centers.^18^ However, to prove that this is the optimal treatment strategy for these patients, evidence from randomized controlled trials must be obtained.

This study has several limitations. First, the study population consisted only of patients who underwent upfront PDAC resection. Patients initially scheduled for pancreatic resection, but who refrained from resection owing to fast progressive disease and health deterioration, were not included. However, the results of this study are still applicable to the vast majority of patients, since approximately 75% of patients scheduled for surgery eventually undergo PDAC resection.^5,44,45^ Second, a complete case analysis based on completeness of “key variables” was performed to allow for a more accurate evaluation of the different staging categories. As a result, patients with unknown preoperative serum CA19-9 or ECOG performance status were excluded, causing potential selection bias. However, comparison of characteristics between included and excluded patients revealed no difference. Moreover, the R_C+_ groups were quite small, so nonsignificant differences may be due to insufficient power. Consequently, the findings with regard to the impact of ECOG performance status as a conditional factor should be interpreted with care. Finally, the current study only included patients with resectable PDAC, as the recommended treatment strategy for borderline resectable PDAC now consists of neoadjuvant therapy instead of upfront resection. Nevertheless, it would be valuable to further explore the impact of biological and conditional factors in patients with borderline resectable PDAC and in the context of neoadjuvant treatment as well, as this could aid in refining patient stratification.

In conclusion, this nationwide observational cohort study demonstrated that patients with anatomically resectable but biologically unfavorable PDAC (defined as a preoperative serum CA19-9 level ≥ 500 U/mL) have worse survival than patients with preoperative serum CA19-9 < 500 U/mL, independent of the patients’ performance status. The inclusion of CA19-9 for preoperative staging of patients with resectable PDAC should be considered, although prospective studies will need to determine whether neoadjuvant treatment is beneficial for these patients.

### Supplementary Information

Below is the link to the electronic supplementary material.

Supplementary file1 (DOCX 569 KB)
