# Supplementary material for: The Value of Biological and Conditional Factors for Staging of Patients with Resectable Pancreatic Cancer Undergoing Upfront Resection: A Nationwide Analysis
Source: Ann Surg Oncol. 2024 Feb 22;31(8):4956–65. doi: 10.1245/s10434-024-15070-w (PMC11236903; doi:10.1245/s10434-024-15070-w)
Supplement: Supplementary file 1 — Supplementary file1 (DOCX 569 KB) [file 10434_2024_15070_MOESM1_ESM.docx]

| **Supplemental Digital Content 1.** Baseline characteristics of 688 patients included in the primary analysis versus 755 patients excluded due to missing key variables who underwent upfront resection of NCCN resectable PDAC between 2014-2019 in the Netherlands^a^ | | | | | |
| --- | --- | --- | --- | --- | --- |
| **Characteristic** | **Eligible for analysis before imputation (n=688)** | **Missing data (%)** | **Excluded due to missing key variables^b^ (n=755)** | **Missing data (%)** | ***P*-value** |
| Male sex, no. (%) | 364 (53) | - | 399 (53) | - | 1.00 |
| Age in years, median (IQR) | 69 (62-74) | - | 69 (62-75) | - | 0.94 |
| BMI, median (IQR) | 24.4 (22.2-27.2) | 4 (1) | 24.4 (22.1-27.2) | 6 (1) | 0.54 |
| Charlson Comorbidity Index, no. (%) |  | 40 (6) |  | 34 (4) | 0.31 |
| < 2 | 427 (66) |  | 457 (63) |  |  |
| ≥ 2 | 221 (34) |  | 267 (37) |  |  |
| ASA classification, no. (%) |  | - |  | 11 (1) | 0.31 |
| I | 65 (9) |  | 79 (11) |  |  |
| II | 436 (63) |  | 442 (59) |  |  |
| III - IV | 187 (27) |  | 223 (30) |  |  |
| ECOG performance status, no. (%) |  | - |  | 402 (53) | 0.33 |
| 0-1 | 621 (90) |  | 311 (88) |  |  |
| 2-4 | 67 (10) |  | 4 (12) |  |  |
| Serum bilirubin (Umol/L), median (IQR) | 31 (10-124) | 136 (20) | 33 (10-111) | 261 (35) | 0.36 |
| Serum CA19-9 (U/mL), median (IQR) | 182 (42-524) | - | 151 (42-520) | 500 (58) | 0.59 |
| Type of surgery, no. (%) |  | 22 (3) |  | 7 (1) | 0.07 |
| Open | 547 (82) |  | 646 (876) |  |  |
| Laparoscopic | 63 (9) |  | 49 (7) |  |  |
| Robotic | 56 (8) |  | 53 (7) |  |  |
| Type of resection, no. (%) |  | 25 (3) |  | 20 (3) | 0.15 |
| Pancreatoduodenectomy | 545 (82) |  | 601 (81) |  |  |
| Distal pancreatectomy | 102 (15) |  | 103 (15) |  |  |
| Total pancreatectomy | 16 (2) |  | 31 (4) |  |  |
| Vascular resection, no. (%) | 165 (24) | - | 178 (24) | 4 (1) | 0.95 |
| Major postoperative complications, no. (%)^c^ | 274 (40) | 2 (0) | 279 (37) | 2 (0) | 0.28 |
| Hospital stay in days, median (IQR) | 10 (8-17) | 116 (17) | 11 (8-17) | 91 (12) | 0.77 |
| 30-day mortality after surgery due to complications, no. (%) | 12 (2) | - | 11 (1) | - | 0.87 |
| Pathologically measured tumor size in cm, no. (%) |  | 19 (3) |  | 20 (3) | 0.41 |
| ≤2 cm | 110 (16) |  | 116 (16) |  |  |
| >2 cm – ≤4 cm | 437 (65) |  | 464 (63) |  |  |
| >4 cm | 122 (18) |  | 155 (21) |  |  |
| 8^th^ AJCC N stage, no. (%) |  | 6 (1) |  | 12 (2) | 0.20 |
| N0 | 177 (26) |  | 208 (28) |  |  |
| N1 | 282 (41) |  | 273 (37) |  |  |
| N2 | 223 (33) |  | 262 (35) |  |  |
| Lymphovascular invasion, no. (%) | 361 (64) | 128 (19) | 396 (64) | 140 (19) | 1.00 |
| Perineural invasion, no. (%) | 506 (87) | 105 (15) | 569 (85) | 86 (11) | 0.42 |
| Resection margin status, no. %) |  | 25 (4) |  | 19 (3) | 0.57 |
| R0 ≥1mm | 336 (51) |  | 363 (49) |  |  |
| R1 <1mm | 327 (49) |  | 372 (51) |  |  |
| R2 macroscopic | 0 (0) |  | 1 (0) |  |  |
| Tumor differentiation, no. (%) |  | 86 (13) |  | 102 (14) | 0.13 |
| Well/moderate | 448 (74) |  | 460 (70) |  |  |
| Poor | 154 (26) |  | 193 (30) |  |  |
| Adjuvant chemotherapy | 404 (62) | 36 (5) | 424 (60) | 52 (7) | 0.57 |
| Percentages may not sum to 100% because of rounding.  ^a^ Patients undergoing neoadjuvant treatment were not considered eligible for inclusion in this study  ^b^ Serum CA19-9, and ECOG performance status were considered key variables  ^c^ Major complications were defined as complications requiring a surgical or radiological intervention, intensive care unit admittance, single- or multi-organ failure or death  AJCC, American Joint Committee on Cancer; ASA, American Society of Anesthesiologists; BMI, body mass index; ECOG, Eastern Cooperative Oncology Group; IQR, interquartile range; NCCN: National Comprehensive Cancer Network; PDAC, pancreatic ductal adenocarcinoma; | | | | | |

**Supplemental Digital Content 2**. Sensitivity analysis: Kaplan-Meier curves and Cox-proportional hazard analysis comparing overall survival between R_B+C+_, R_B+C-_, R_B-C+_, and R_B-C-_ PDAC, stratified for **(A)** patients with hyperbilirubinemia **(B)** patients without hyperbilirubinemia.

**
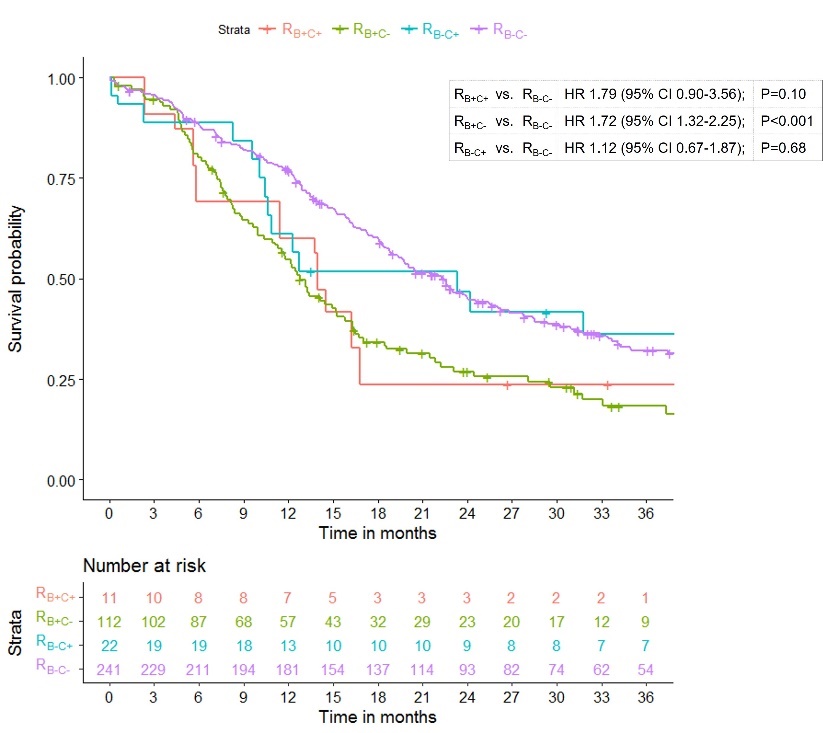
**

**A**

**
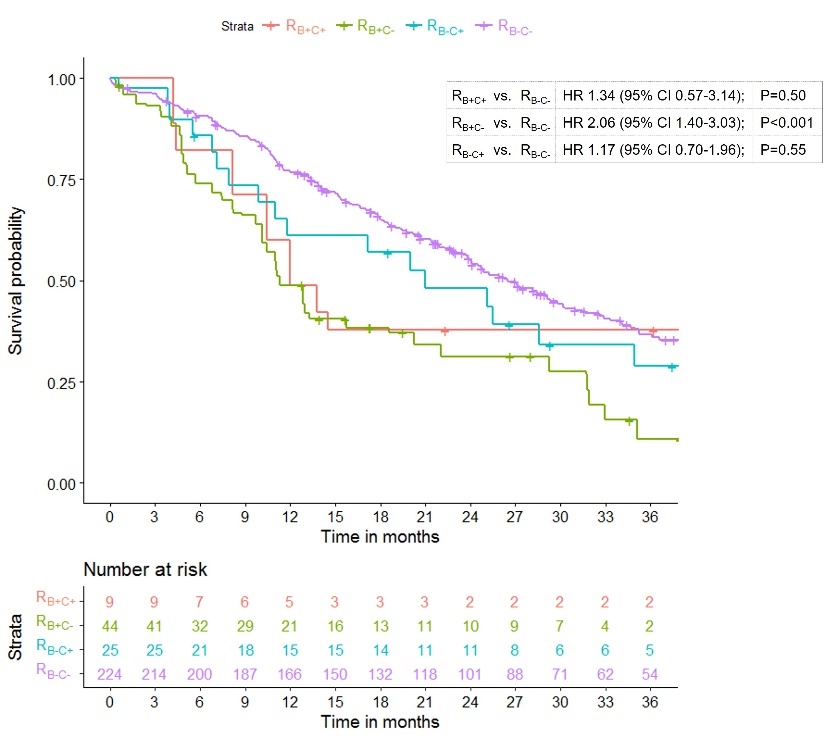
**

**B**

R_B+_ was defined as preoperative serum CA19-9 levels ≥500 U/mL, and R_B-_ as CA19-9 <500 U/mL. R_C+_ was considered with an ECOG performance status ≥2, and R_C-_ with ECOG 0-1.

**Supplemental Digital Content 3**. Sensitivity analysis: Kaplan-Meier curves and Cox-proportional hazard analysis comparing overall survival between patients with R_B+C+_, R_B+C-_, R_B-C+_, and R_B-C-_ PDAC in patients secreting CA19-9 (i.e. serum CA19-9 levels ≥5 U/mL).

**
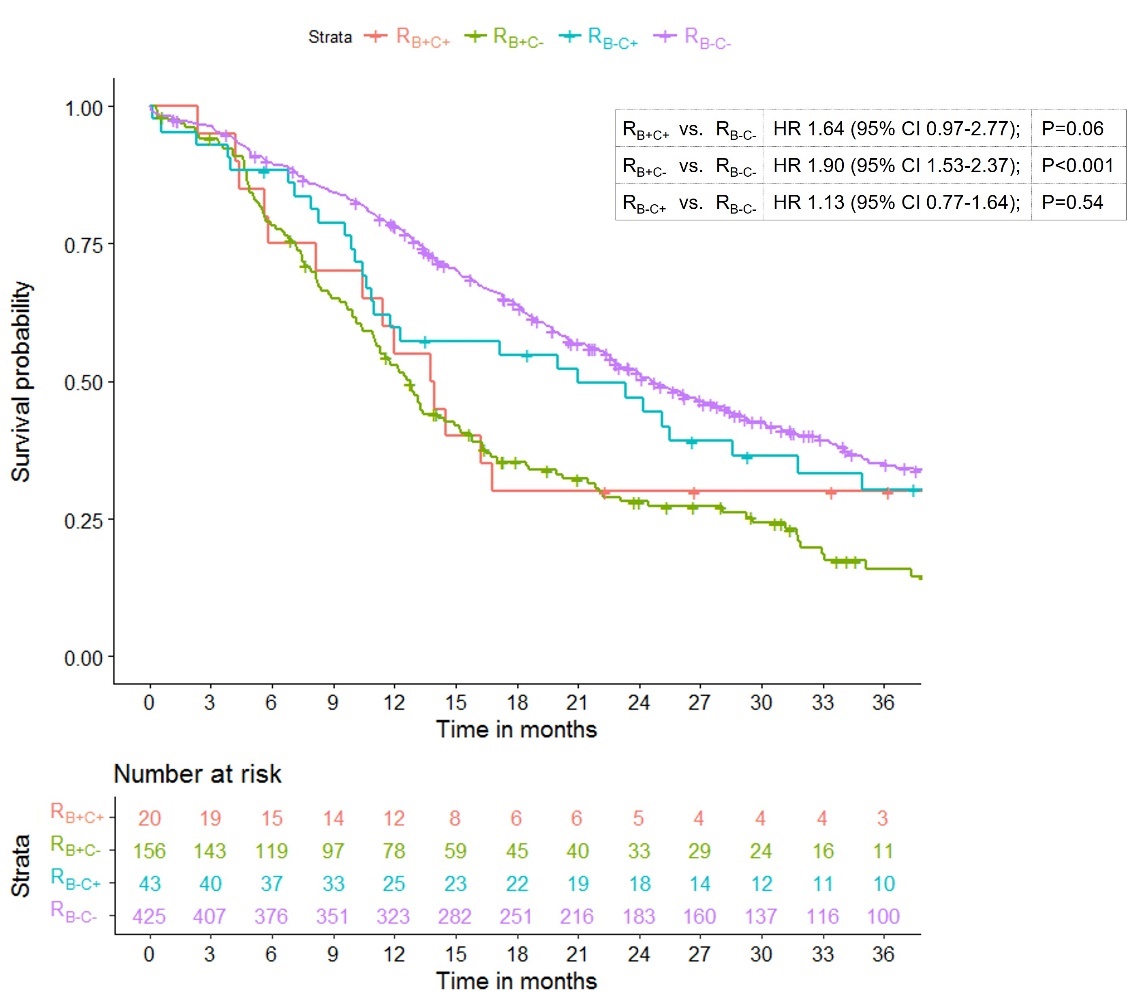
**

R_B+_ was defined as preoperative serum CA19-9 levels ≥500 U/mL, and R_B-_ as CA19-9 <500 U/mL. R_C+_ was considered with an ECOG performance status ≥2, and R_C-_ with ECOG 0-1.

**Supplemental Digital Content 4**. Sensitivity analysis: Kaplan-Meier curves and Cox-proportional hazard analysis comparing overall survival between patients with R_B+C+_, R_B+C-_, R_B-C+_, and R_B-C-_ PDAC, defining R_C+_ as Eastern Cooperative Oncology Group performance status ≥1.


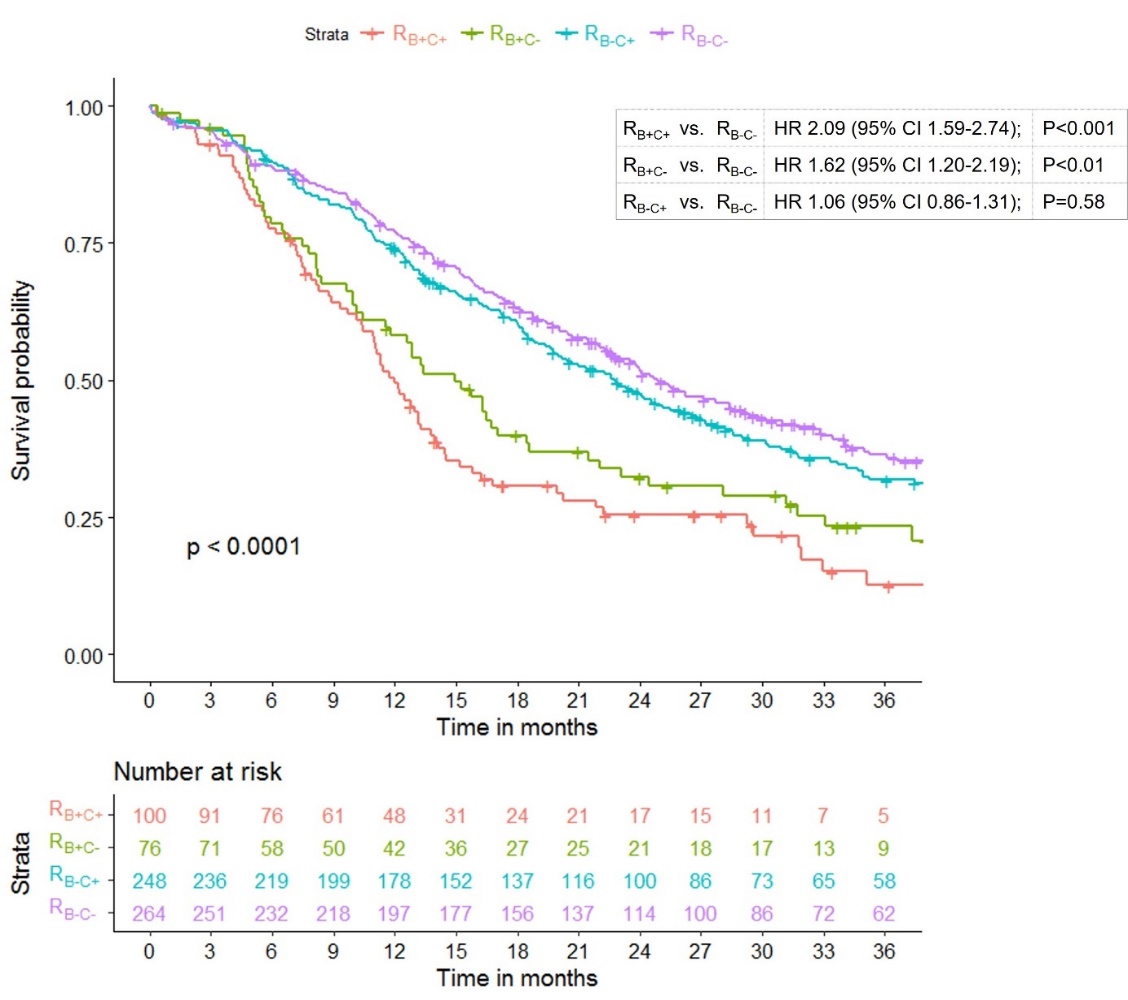


R_B+_ was defined as preoperative serum CA19-9 levels ≥500 U/mL, and R_B-_ as CA19-9 <500 U/mL. R_C+_ was considered with an ECOG performance status ≥1, and R_C-_ with ECOG 0.
